# Enhancing CO_2_ electroreduction to CH_4_ over Cu nanoparticles supported on N-doped carbon†

**DOI:** 10.1039/d2sc02222b

**Published:** 2022-07-05

**Authors:** Yahui Wu, Chunjun Chen, Xupeng Yan, Ruizhi Wu, Shoujie Liu, Jun Ma, Jianling Zhang, Zhimin Liu, Xueqing Xing, Zhonghua Wu, Buxing Han

**Affiliations:** Beijing National Laboratory for Molecular Sciences, CAS Key Laboratory of Colloid and Interface and Thermodynamics, CAS Research/Education Center for Excellence in Molecular Sciences, Institute of Chemistry, Chinese Academy of Sciences Beijing 100190 P. R. China hanbx@iccas.ac.cn chenchunjun@iccas.ac.cn; University of Chinese Academy of Sciences Beijing 100049 China; Chemistry and Chemical Engineering of Guangdong Laboratory Shantou 515063 China; Physical Science Laboratory, Huairou National Comprehensive Science Center Beijing 101400 China; Shanghai Key Laboratory of Green Chemistry and Chemical Processes, School of Chemistry and Molecular Engineering, East China Normal University Shanghai 200062 China; Institute of High Energy Physics, Chinese Academy of Sciences Beijing 100049 China

## Abstract

The electroreduction of CO_2_ to CH_4_ has attracted extensive attention. However, it is still a challenge to achieve high current density and faradaic efficiency (FE) for producing CH_4_ because the reaction involves eight electrons and four protons. In this work, we designed Cu nanoparticles supported on N-doped carbon (Cu-np/NC). It was found that the catalyst exhibited outstanding performance for the electroreduction of CO_2_ to CH_4_. The FE toward CH_4_ could be as high as 73.4% with a high current density of 320 mA cm^−2^. In addition, the mass activity could reach up to 6.4 A mg_Cu_^−1^. Both experimental and theoretical calculations illustrated that the pyrrolic N in NC could accelerate the hydrogenation of *CO to the *CHO intermediate, resulting in high current density and excellent selectivity for CH_4_. This work conducted the first exploration of the effect of N-doped species in composites on the electrocatalytic performance of CO_2_ reduction.

## Abstract 2

The pyrrolic N in Cu-np/NC composites can enhance the activation of H_2_O, resulting in excellent selectivity for CH_4_ and high current density.

## Graphical abstract

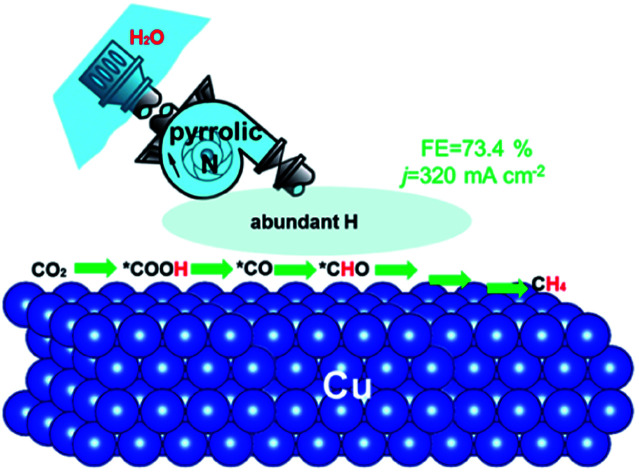

## Introduction

The electrochemical CO_2_ reduction reaction (CO_2_RR) is a promising approach to achieve carbon neutrality,^1–6^ which can convert CO_2_ into valuable chemicals and fuels using renewable energy.^7–11^ Among all these products, CH_4_ is a highly desired product because it holds the highest heating value of 55.5 MJ kg^−1^.^12^ However, CO_2_ electroreduction to CH_4_ still suffers from high overpotential and low activity and selectivity.^13–16^ Designing highly efficient and robust electrocatalysts is crucial to solve this problem.

Cu-based catalysts have been proven to be the most promising electrocatalysts for producing hydrocarbon products from the CO_2_RR. In recent years, many methods have been applied to enhance the activity of the CO_2_RR over Cu-based catalysts, including alloying,^17^ doping,^18,19^ modifying with other compounds,^20,21^ changing the shape and size,^22–24^ and building an interface and defects.^25–27^ However, it is still challenging to achieve high selectivity for CH_4_ at a high current density.^28–33^ This is because the generation of CH_4_ involves eight electrons and four protons, which easily bifurcates to give broad product distributions.^34–36^ According to a previous report,^37^ the co-adsorption of *CO and *H played an important role in the production of CH_4_, and the selectivity of CH_4_ can be enhanced by a high surface *H coverage, due to sufficient *H supply for the hydrogenation of intermediates.^37^ However, the high surface *H coverage could result in the undesired hydrogen evolution reaction (HER) and hinder the adsorption of intermediates. Thus, it is necessary to find a method to break the linear scale relationship of the single catalyst sites, which will enhance the selectivity of CH_4_ and decrease the yield of H_2_ simultaneously.

To solve the above problem, here we proposed the idea to introduce additional catalytic sites, which can enhance the activation of H_2_O and hydrogenation of intermediates but not cause excessive production of H_2_. According to a previous report, N-doped carbon (NC) catalysts could promote the activation of H_2_O.^38–40^ In addition, the adsorption of *H can be regulated by changing the type of N-doped species.^41–43^ Thus we can assume that NC would be a potential platform for tuning the activity and selectivity of Cu-based catalysts. Although NC has been used to modify Cu-based catalysts, the products were ethanol and ethylene.^41^ In addition, the role of the N-doped species in the composites is not clear.

Herein, we used NC with different contents of N-doped species as another component to modify Cu nanoparticles (Cu-np/NC). In this strategy, the selectivity of CH_4_ over Cu-np could be enhanced by introducing NC with rich pyrrolic N species, and the faradaic efficiency (FE) of CH_4_ could reach up to 73.4% with a current density of 320 mA cm^−2^. Especially, the mass activity reached up to 6.4 A mg_Cu_^−1^. An *in situ* surface enhanced Raman spectroscopy (SERS) study demonstrated that the formation of the *CHO intermediate could be promoted over Cu-np/NC, which is an important intermediate for producing CH_4_. Experimental and DFT studies indicated that the pyrrolic N in NC could accelerate the hydrogenation of intermediates, resulting in excellent selectivity for CH_4_.

## Results and discussion

Cu-np and NC were prepared respectively according to previous reports.^44,45^ The size of the obtained Cu-np was about 40 nm (Fig. S1†). NC(*x* : *y*) was prepared using arginine and melamine,^45^ and *x* : *y* represents the ratio of arginine and melamine in the preparation process. All of the NC(*x* : *y*) exhibited a nanosheet morphology (Fig. S2†). Cu-np/NC(*x* : *y*) was prepared by blending Cu-np and NC(*x* : *y*) (see the Methods for experimental details).

The electrocatalytic performance of Cu-np/NC(1 : 4) was first studied, and the other Cu-np/NC(*x* : *y*) catalysts will be studied in the following section. The electrocatalytic performance of the CO_2_RR was evaluated in a flow cell, as reported in our previous report.^34,41^ The catalysts were sprayed on hydrophobic carbon paper as the cathode, and Ni foam was used as the anode.^34,41,46,47^ 1 M KOH solution was used as the electrolyte. The gaseous and liquid products were analyzed by gas chromatography (GC) and nuclear magnetic resonance (NMR) spectroscopy, respectively.

**Fig. 1:**
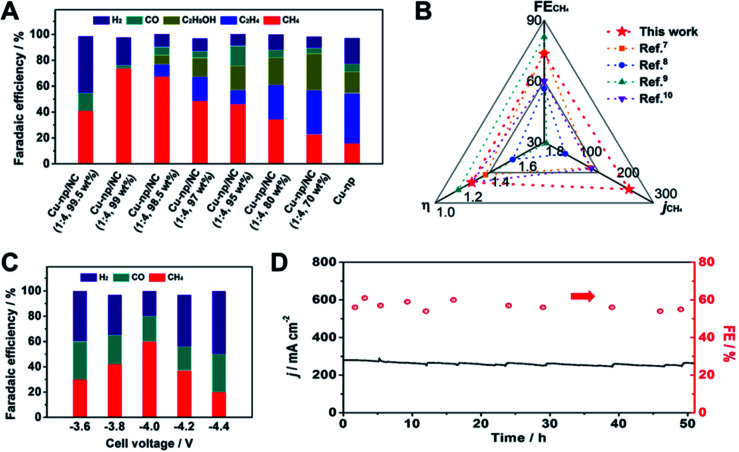
(A) The distribution of products at −1.1 V *vs.* RHE over Cu-np and Cu-np/NC(1 : 4). (B) Comparison of the overpotential (*η*), FE and CH_4_ partial current density of Cu-np/NC(1 : 4, 99 wt%) with those of state-of-the-art Cu-based catalysts. (C) The distribution of products over Cu-np/NC(1 : 4, 99 wt%) at different cell voltages in a MEA system. (D) The current density and FE of CH_4_ on Cu-np/NC(1 : 4, 99 wt%) at −4 V in 50 hour potentiostatic electrolysis tests.

Over Cu-np, CO_2_ could be reduced to various products, such as CO, CH_4_, C_2_H_4_ and C_2_H_5_OH (Fig. 1A and S3†). However, the selectivity of a single product is low (less than 45%). It is noted that the selectivity of CH_4_ was significantly enhanced by adding NC(1 : 4) and increased with the content of NC(1 : 4) (Fig. 1A and S4†). The selectivity of CH_4_ reached the highest when the content of NC(1 : 4) was 99 wt% (Cu-np/NC(1 : 4, 99 wt%)). The FE of CH_4_ could reach up to 73.4%, which is much higher than that of Cu-np. When the content of NC(1 : 4) was 99.5 wt%, the selectivity of CH_4_ decreased with increasing yield of H_2_, which may have originated from the insufficient Cu sites. For NC(1 : 4), only small amounts of CO were detected (Fig. S5†). Consequently, it can be assumed that the Cu sites were the active sites and the selectivity of CH_4_ was enhanced by the addition of NC(1 : 4). The partial current density of CH_4_ over Cu-np/NC(1 : 4, 99 wt%) was 234 mA cm^−2^ at −1.1 V *vs.* RHE, which is about 4.1 times higher than that over Cu-np (Fig. S6†). In the meantime, the CH_4_-to-other ratio was enhanced from 0.25 on Cu-np to 20.2 on Cu-np/NC(1 : 4, 99 wt%) (Fig. S7†), indicating that the selectivity of CH_4_ was enhanced and the other products were suppressed. Compared with the state-of-the-art catalysts, Cu-np/NC(1 : 4, 99 wt%) performed as one of the best catalysts in FE, current density and overpotential for CH_4_ (Fig. 1B and Table S1†). In addition, based on such a low content (1 wt%) of Cu-np in Cu-np/NC(1 : 4, 99 wt%), the mass activity could be as high as 6.4 A mg_Cu_^−1^. In the following discussion, the content of NC was fixed at 99 wt% in Cu-np/NC(*x* : *y*).

The electrolysis of the CO_2_RR was also carried out using membrane electrode assembly-based reactors (Fig. S8†). Cu-np/NC(1 : 4) was used as the cathode catalyst for the CO_2_RR and an iridium oxide-based catalyst as the anode for the oxygen evolution reaction. The selectivity of CH_4_ was 60% with a current density of 230 mA cm^−2^ at a cell voltage of −4 V (Fig. 1C and S9†). Furthermore, the stability of Cu-np/NC(1 : 4) was investigated at a cell voltage of −4 V. H_2_O was injected into the cathode flow channel to prevent salt accumulation in the gas diffusion layer (GDL) micropores. The selectivity of CH_4_ and the current density had no obvious change for 50 h (Fig. 1D), indicating that Cu-np/NC(1 : 4) exhibited excellent stability.

The electrocatalytic performance of the CO_2_RR over other Cu-np/NC(*x* : *y*) was also evaluated in a flow cell. As shown in Fig. S10,† the selectivity of CH_4_ over Cu-np/NC(*x* : *y*) varied with the *x* : *y* value. The highest FEs of CH_4_ over Cu-np/NC(1 : 2) and Cu-np/NC(1 : 8) were 65.8% and 59.6% respectively. Thus, we can deduce that NC(*x* : *y*) played an important role in the selectivity of CH_4_. According to a previous report,^45^ the intrinsic properties of NC were mainly attributed to the N-doped species. The correlation between CH_4_ selectivity and the type of N-doped species was investigated (Fig. S11, S12 and Table S2†). The FE of CH_4_ increased with increasing pyrrolic N content, whereas no regularity can be found for pyridinic N and graphitic N. These results indicated that the pyrrolic N in the NC(*x* : *y*) may play a crucial role in the enhancement of selectivity of CH_4_.

In addition, the electrochemically active surface areas (ECSAs) and Nyquist plots of Cu-np/NC(*x* : *y*) were measured. The charge transfer resistance (Rct) for the different Cu-np/NC(*x* : *y*) was similar (Fig. S13†). Although the ECSAs of Cu-np/NC(*x* : *y*) varied slightly with the different NC(*x* : *y*) (Fig. S14†), the normalized partial current densities for CH_4_ by ECSAs were similar to the geometric partial current density (Fig. S15†). These results indicated that different CO_2_RR performances of Cu-np/NC(*x* : *y*) with different *x* : *y* values were not originated from the slight change of the *R*_ct_ and ECSAs.

**Fig. 2:**
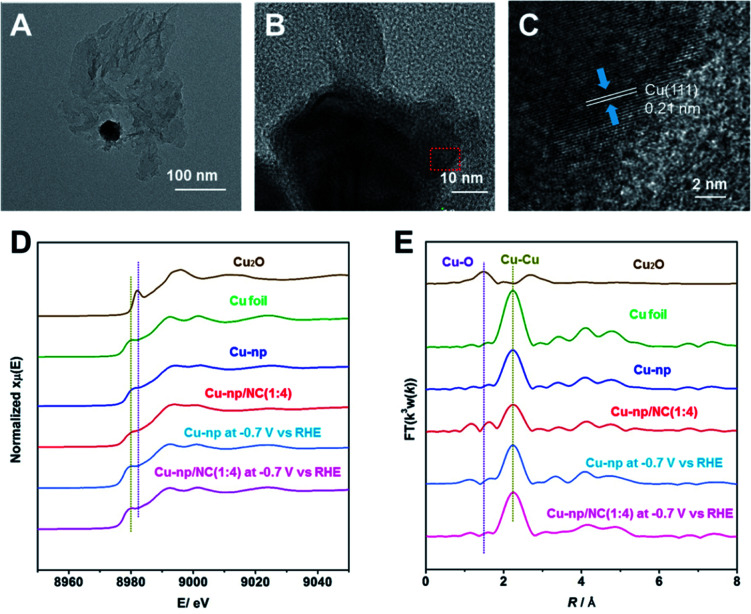
(A and B) The TEM images of Cu-np/NC(1 : 4). (C) The HR-TEM image of Cu-np/NC(1 : 4). (D) The operando XANES spectra at the Cu K-edge for Cu-np and Cu-np/NC(1 : 4) at −0.7 V *vs.* RHE during the CO_2_RR. (E) The corresponding Fourier transform FT(k^3^w(k)) for Cu-np and Cu-np/NC(1 : 4) at −0.7 V *vs.* RHE during the CO_2_RR.

Cu-np/NC(1 : 4) was characterized by transmission electron microscopy (TEM), and we can observe that Cu-np was dispersed on NC(1 : 4), as shown in Fig. 2A and B. In addition, the lattice distance of Cu(111) was observed by high-resolution transmission electron microscopy (HR-TEM) (Fig. 2C), which is consistent with that in Cu-np. However, the characteristic peaks of Cu cannot be observed on Cu-np/NC(1 : 4) in XRD patterns, and this is because the content of Cu is too low (Fig. S16†).

X-ray photoelectron spectroscopy (XPS) and operando X-ray absorption spectroscopy (XAS) were carried out to monitor the valence state and coordinate environment of Cu during the CO_2_RR by the method used in our previous study.^34,41,48^ From XPS, we can observe that the Cu valence state in Cu-np and Cu-np/NC(1 : 4) was similar, which is attributed to Cu^0^ (Fig. S17†). As shown in X-ray absorption near edge structure (XANES) spectroscopy (Fig. 2D), the pre-edge peaks of Cu-np and Cu-np/NC(1 : 4) were close to Cu foil before reaction. When the potential (−0.7 V *vs.* RHE) was applied, the spectra of Cu-np and Cu-np/NC(1 : 4) were still similar to that of metallic Cu. According to extended X-ray absorption fine structure (EXAFS) spectroscopy (Fig. 2E and S18†), only a peak corresponding to the Cu–Cu bond was observed, indicating that the metallic Cu was the active site for Cu-np and Cu-np/NC(1 : 4) during the CO_2_RR. Furthermore, the Cu–Cu coordination number of Cu-np and Cu-np/NC(1 : 4) during the CO_2_RR were quantified by using the ARTEMIS programs of IFEFFIT (Fig. S19, S20 and Table S3†).The Cu–Cu coordination number and bond distance in Cu-np/NC(1 : 4) were close to that in Cu-np during the CO_2_RR. These results indicate that the addition of NC did not change the coordination properties of Cu during the CO_2_RR.

DFT calculations were then carried out to gain insights into the effect of the N-doped species in Cu-np/NC on the selectivity of CH_4_. Cu(111) was used to represent Cu-np (Fig. S21†), which is in accordance with the results of HR-TEM. Cu(111) was located on a layer of N-doped graphene (NG) to represent the model of Cu-np/NC (Fig. S22†). From the results above, the pyrrolic N species played a crucial role in the selectivity of CH_4_. Then, the reaction energy diagrams of CO_2_ reduction to CH_4_ were first characterized over Cu(111) and Cu(111) on pyrrolic N-doped graphene (Cu(111)/pyrrolic N).

As shown in Fig. 4A, S23 and S24,† CO_2_ was first reduced to *CO through the *COOH intermediate, and then *CO was further reduced to CH_4_ through the *CHO intermediate. On Cu(111), the hydrogenation of *CO to *CHO shows the highest energy barrier (0.71 eV), which is considered as the rate-limiting step for producing CH_4_. The hydrogenation of CO_2_ to *COOH and hydrogenation of *CO to *CHO were promoted over Cu(111)/pyrrolic N. Although the hydrogenation of *CO to *CHO still shows the highest energy barrier over Cu(111)/pyrrolic N, it was only 0.30 eV, which was much lower than that over Cu(111). These results suggested that the reduction of CO_2_ to CH_4_ over Cu(111) can be significantly enhanced by combining with pyrrolic N doped NC. Furthermore, the reaction energy diagrams were characterized at −0.5 V applied potential (Fig. S25†), and Cu(111)/pyrrolic N also is more favorable for producing CH_4_ than Cu(111).

**Fig. 3:**
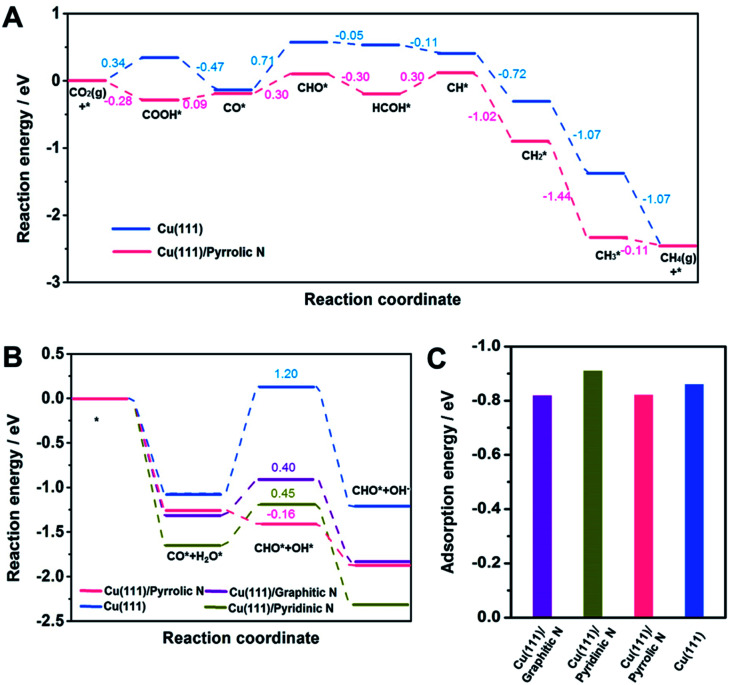
(A) A reaction energy diagram for the CO_2_RR to CH_4_ over Cu(111) and Cu(111)/pyrrolic N. (B) A reaction energy diagram for *CO hydrogenation to *CHO on Cu(111), Cu(111)/graphitic N, Cu(111)/pyridinic N and Cu(111)/pyrrolic N. (C) The adsorption energy of *CO on different models.

In addition, the hydrogenation of *CO to *CHO was also studied over Cu (111)/NC with different N-doped species (Fig. 3B and S26–S29†), and H_2_O was used as the donor of hydrogen, because 1 M KOH solution was used as the electrolyte in the CO_2_RR. Compared with Cu(111), the formation of *CHO and *OH from *CO and *H_2_O can be enhanced over Cu(111)/graphitic N, Cu(111)/pyrrolic N and Cu (111)/pyridinic N. It is noted that Cu(111)/pyrrolic N exhibited the lowest energy barrier (−0.16 eV), indicating that the pyrrolic N played the main role in the outstanding activity and selectivity of CH_4_, which was consistent with the experimental results.

According to a previous report,^49^ the adsorption of *CO played an important role in the selectivity of products. Thus, the adsorption of *CO on different models was studied. The adsorption of *CO on Cu(111), Cu(111)/graphitic N, Cu(111)/pyrrolic N and Cu(111)/pyridinic N was comparable (Fig. 3C), indicating that the adsorption of *CO was not changed by adding NC. Thus we can assume that the outstanding performance for CH_4_ over Cu-np/NC was attributed to the activation of H_2_O by pyrrolic N.

**Fig. 4:**
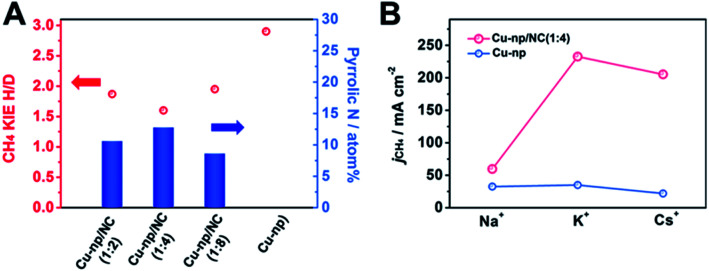
(A) Kinetic isotopic effect (KIE) of H/D on the CO_2_RR to CH_4_ at −1.1 V *versus* RHE. (B) Effect of alkali metal cations in the MOH (M = Na^+^, K^+^ and Cs^+^) electrolyte on the CO_2_RR to CH_4_ at −1.1 V *versus* RHE over Cu-np and Cu-np/NC(1 : 4) catalysts.

From the results of DFT calculations above, we can know that the pyrrolic N in NC can enhance the activation of H_2_O and accelerate the hydrogenation of intermediates. To explore the effect of H_2_O activation on the generation of CH_4_, the kinetic isotopic effect (KIE) of H/D over Cu-np/NC(*x* : *y*) catalysts was measured (Fig. 4A and S30†). The KIEs of H/D are defined as the ratio of CH_4_ formation rates in H_2_O and D_2_O. It has been reported that the reaction is considered to be controlled by the primary isotope effect when the KIE value is greater than 2.^49,50^ The KIE value was 2.9 over Cu-np, suggesting that the activation of H_2_O was involved in the rate-determining step. The KIE value over Cu-np/NC(*x* : *y*) decreased with the increase of the content of pyrrolic N in NC, suggesting that the dissociation of H_2_O can be enhanced by the pyrrolic N. For Cu-np/NC(1 : 4), the content of pyrrolic N reached the highest, and the KIE value was about 1.6, indicating that the dissociation of H_2_O was no longer involved in the rate-determining step, which is consistent with the results of DFT. Thus it can be deduced that the pyrrolic N in NC can accelerate H_2_O activation.

The role of H_2_O activation in the generation of CH_4_ was further studied by investigating the effect of alkali metal (M) cations in a MOH electrolyte. It is known that the cation can combine with H_2_O to form a hydrated cation of M^+^(H_2_O)*n*, and the value of *n* was 13, 7 and 6 for Na^+^, K^+^ and Cs^+^, respectively.^49^ The radii of M^+^(H_2_O)*n* decrease in the order of Na^+^ > K^+^ > Cs^+^.^51^ The smaller *n* and radii of M^+^(H_2_O)*n* enable a greater ability to dissociate H_2_O.^49,51^ It can be known that the formation rate of CH_4_ was improved markedly over Cu-np/NC(1 : 4) by changing the cation from Na^+^ to K^+^ (Fig. 4B). The formation rate of CH_4_ in CsOH was smaller than that in KOH. This may be because the generation of H_2_ was also enhanced (Fig. S31†). For Cu-np, the formation rate of CH_4_ increased slightly when changing the cation from Na^+^ to K^+^. Thus we can assume that NC can enhance CH_4_ formation by promoting H_2_O activation through interaction with hydrated cations.

**Fig. 5:**
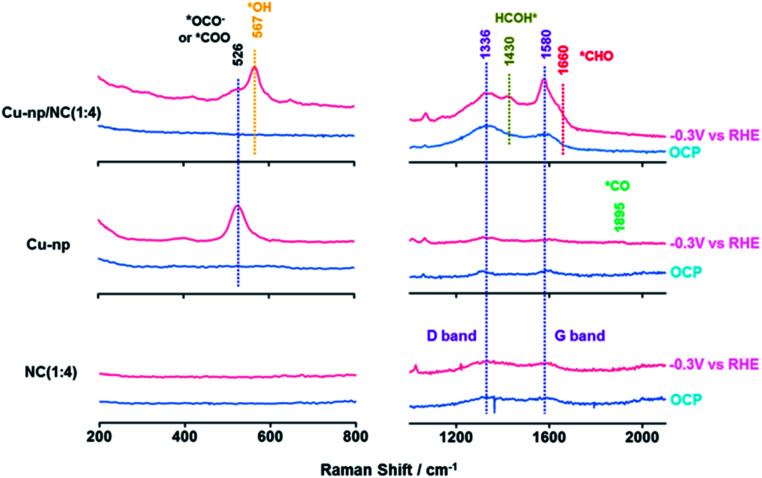
The *in situ* surface-enhanced Raman spectra over Cu-np, Cu-np/NC(1 : 4) and NC(1 : 4) at −0.3 V *vs.* RHE during the CO_2_RR.

Furthermore, the reaction intermediates during the CO_2_RR were traced by *in situ* surface-enhanced Raman spectroscopy (SERS).^34,41^ At open-circuit potential (OCP), no Cu_*x*_O species were observed on Cu-np and Cu-np/NC(1 : 4), which was consistent with the results of XPS and XAS. The peaks located at 1336 cm^−1^ and 1580 cm^−1^ were observed on NC and Cu-np/NC(1 : 4), which were assigned to the D band and G band of graphene, respectively.^52–54^ Weak peaks were also observed on Cu-np, which may be from the carbon paper. It is noted that a new Raman peak located at 526 cm^−1^ appeared on Cu-np and Cu-np/NC(1 : 4) at −0.3 V *vs.* RHE, which was attributed to the adsorption of preliminary intermediates (such as *CO_2_ or *OCO^−^) on the Cu surface.^55^ These results indicated that the activation of CO_2_ occurred on Cu sites. For Cu-np, a new Raman band located at 1895 cm^−1^ appeared at −0.3 V *vs.* RHE, which corresponded to the C≡O stretching on Cu.^41^ In contrast, no adsorption of *CO was observed on Cu-np/NC(1 : 4). This may be because the obtained *CO can be consumed quickly. Compared with Cu-np, Cu-np/NC(1 : 4) showed three other new peaks located at 567 cm^−1^, 1430 cm^−1^ and 1660 cm^−1^ at −0.3 V *vs.* RHE, which may be attributed to the adsorption of *OH, *HCOH and *CHO on the Cu surface.^50,54,55^ Thus we can deduce that the reaction *CO + *H_2_O → *CHO + *OH can be accelerated over Cu-np/NC and thus the selectivity of CH_4_ can be enhanced, which was consistent with the experimental and calculation results (Fig. 5).

## Conclusions

In summary, Cu-np/NC composites were designed to enhance the electroreduction of CO_2_ to CH_4_. The selectivity of CH_4_ reached 73.4%, with a current density of 320 mA cm^−2^. Based on experimental and theoretical studies, the effect of the N-doped species on the electrocatalytic performance of CO_2_ reduction was elucidated. The pyrrolic N in NC can enhance the activation of H_2_O, which could accelerate the hydrogenation of intermediates, resulting in excellent selectivity for CH_4_ and high current density. We believe that this work opens a way for the design of efficient catalysts for the electroreduction of CO_2_ to CH_4_.

## Data availability

The data that support the findings of this study are available within the article and its ESI.†

## Author contributions

Y. H. W., C. J. C., and B. X. H. proposed the project, designed the experiments, and wrote the manuscript; Y. H. W. performed the whole experiments; X. P. Y., R. Z. W., S. J. L., J. M., J. L. Z., Z. M. L., X. Q. X., Z. H. W. performed the analysis of experimental data; C. J. C. and B. X. H. supervised the whole project.

## Conflicts of interest

There are no conflicts to declare.

## Supplementary Material

SC-013-D2SC02222B-s001
