# Supplementary material for: Enhancing CO2 electroreduction to CH4 over Cu nanoparticles supported on N-doped carbon
Source: Chem Sci. 2022 Jul 5;13(28):8388–94. doi: 10.1039/d2sc02222b (PMC9297438; doi:10.1039/d2sc02222b)
Supplement: SC-013-D2SC02222B-s001 [file SC-013-D2SC02222B-s001.pdf]

Electronic Supplementary Material (ESI) for Chemical Science.
This journal is © The Royal Society of Chemistry 2022

WSupporting Information

Enhancing CO_2 electroreduction to CH_4 over the Cu nanoparticles supported on N-doped carbon

Yahui Wu,[a,b] Chunjun Chen,[a,b*] Xupeng Yan,[a,b] Ruizhi Wu,[a,b] Shoujie Liu,[c] Jun Ma,[a] Jianling Zhang,[a] Zhimin Liu,[a, b] Xueqing Xing,[f] Zhonghua Wu,[f] Buxing Han,[a,b,d,e] *

[a] Beijing National Laboratory for Molecular Sciences, Key Laboratory of Colloid and Interface and Thermodynamics, Institute of Chemistry, Chinese Academy of Sciences, Beijing 100190, P. R. China

[b]School of Chemistry and Chemical Engineering, University of Chinese Academy of Sciences,Beijing 100049, China

[c]Chemistry and Chemical Engineering of Guangdong Laboratory, Shantou 515063, China

[d]Physical Science Laboratory, Huairou National Comprehensive Science Center, No. 5 Yanqi East Second Street, Beijing 101400, China

[e]Shanghai Key Laboratory of Green Chemistry and Chemical Processes, School of Chemistry and Molecular Engineering, East China Normal University, Shanghai 200062, China

[f]Institute of High Energy Physics, Chinese Academy of Sciences, Beijing 100049, China

Experimental Section

Materials: Copper(II) Acetylacetonate ($Cu(acac)_2$ ≥ 98.0%), Oleylamine ($C_{18}H_{37}N$, OAm 80%~90%), potassium hydroxide (KOH, ≥85%), L-Ascorbic acid ($C_6H_8O_6$, ≥ 99%) and sodium 2, 2-dimethyl-2-silapentane-5-sulfonate (DSS, 99%) were purchased from Sigma-Aldrich. Cesium hydroxide monohydrate ($CsOH \cdot H_2O$, 95%), sodium hydroxide (NaOH, ≥ 99.0%) and Ni foam were provided by Sinopharm Chemical Reagent Co. Ltd. D_2O (98%), L-Arginine ($C_6H_{14}N_4O_2$, 98%), melamine ($C_3H_6N_6$, 99%) were obtained from Beijing Innochem Science & Technology Co. Ltd. All the chemicals were used as received. N_2 (99.999%) and CO_2 (99.999%) were provided by Beijing Analytical Instrument Company. Deionized water was used in the experiments.

Synthesis of Cu-np: According to previous report,[S1] $Cu(acac)_2$ (44.0 mg), L-Ascorbic acid (210.0 mg) were pre-dissolved in OAm (20 mL) in 50 mL vial. The mixture was sonicated for 15 min and transferred to an oil bath, which was heated at 130°C for 4 h and cooled to room temperature. The synthesized colloidal products were washed five times with hexane/ethanol solvents and collected by centrifuge at 9500 rpm，Finally obtained samples were dispersed in 5 mL methanol and stored in the glove box. The content of the Cu-np in the methanol was measured by ICP.

Synthesis of NC (x:y): According to previous literature report,[S2] melamine would transfer to pyridinic-N and graphitic-N. Otherwise, quenching process of arginine produces significant amount of N-H bond, which is contribute to the formation of pyrrolic-N. In a typical procedure, arginine and melamine with various mass ratios (x:y= 1:2, 1:4 and 1:8) were first thoroughly grounded in an agate mortar. The mixed powders were put into a porcelain boat and then transferred to a tube furnace. The mixture was first heated to 650 °C in N_2 atmosphere at a ramp rate of 5 °C min^{-1}. After keeping at 650 °C for 2 h, the sample was cooled down naturally to room temperature, the resultant black product was grounded into powder using an agate mortar and was used for the preparation of catalyst.

Synthesis of Cu-np/NC (x wt%): The Cu-np/NC was synthesized by simple ultrasonic treatment. In a typical procedure, 25 mg NC was dispersed in 1 mL of ethanol, then various content of Cu-np

was added under ultrasound for 0.5 hours. The solution can be directly used for the preparation of electrode.

Materials characterizations: The microstructures of the catalysts were characterized by scanning electron microscope (SEM, HITACHI S-4800) and transmission electron microscopy (TEM, JEOL JEM-2100F) equipped with EDS. X-ray photoelectron spectroscopy (XPS) study was carried out on the Thermo Scientific ESCALab 250Xi using a 200W Al-Kα radiation. In the analysis chamber, the base pressure was about 3×10^{-10} mbar. Typically, the hydrocarbon C1s line at 284.8 eV from adventitious carbon was used for energy referencing. Raman spectroscopy (Horiba Labram HR Evolution Raman System) was conducted using a 785-nm excitation laser. The operando X-ray absorption spectroscopy (XAS) measurements were performed using a modified flow cell at the line at the 1W1B, 1W2B and 4B9A beamline at Beijing Synchrotron Radiation Facility (BSRF), China. In situ Raman measurements were carried out using a LabRAM HR Evolution Raman microscope in a modified flow cell. A 785-nm laser was used and signals were recorded using a 20 s integration and by averaging two scans.

Preparation of electrodes. To construct the cathode electrode, the obtained solution of Cu-np/NC (0.25 mL) was sprayed on the hydrophobic carbon paper (YLS-30T) to achieve a catalyst loading of ~5.0 mg cm^{-2}. Ni foam was used as anode electrode.

Electrochemical study. Electrochemical studies were conducted in an electrochemical flow cell including a gas chamber, a cathodic chamber, and an anodic chamber, which was similar to that reported.[S3] An anion exchange membrane (FumasepFAA-3-PK-130) was used to separate the anodic and cathodic chambers, and an Ag/AgCl electrode and Ni foam were used as the reference and counter electrodes, respectively. The electrolysis was conducted using a CHI 660e electrochemical workstation equipped with a high current amplifier CHI 680c.

The measured potentials after iR compensation were rescaled to the RHE by E (versus RHE)= E (versus Ag/AgCl)+ 0.197 V+0.0591V/pH×pH. For performance studies, 1 M KOH was used as the electrolyte, and it was circulated through the cathodic and anodic chambers using peristaltic pumps at a rate of 20 mL min^{-1}. The flow rate of CO_2 gas through the gas chamber was controlled to be 20 sccm using a digital gas flow controller. The kinetic isotopic effect (KIE) experiment was conducted in the 1 M KOH solution, in which H_2O was replaced by D_2O.

For the membrane electrode assembly-based reactors, an anion-exchange membrane (Dioxide Materials and Membranes International) was used for cation exchange. Around 5 mg cm^{-2} Cu-np/NC(1:4) loaded onto the YLS-30T GDL electrode (4 cm^2 electrode area) was used as the cathode, and IrO_2 loaded onto a titanium mesh as the anode. The cathode side was supplied with 50 cm^3 min^{-1} humidified CO_2 gas, and 0.1 M $KHCO_3$ aqueous solution was circulated around the anode side at 20 ml min^{-1}.

EIS study. The EIS measurement was carried out in 1 M KOH solution at an open circuit potential (OCP) with an amplitude of 5 mV of 10^{-2} to 10^6 Hz.

Double-layer capacitance (C_{dl}) measurements. The electrochemical active surface area is proportional to C_{dl} value. Cdl was determined by measuring the capacitive current associated with double-layer charging from the scan-rate dependence of cyclic voltammogram (CV). The CV ranged from -0.0 V to -0.1 V vs. RHE. The Cdl was estimated by plotting the Δj (j_a -j_c) at -0.05 V vs. RHE against the scan rates, in which the j_a and j_c were the anodic and cathodic current density, respectively. The scan rates were 20, 30, 40, 50, 60, 70, 80, 90, 100, 110 and 120 mV s^{-1} .

Product analysis. The gaseous product of electrochemical experiments was collected using a gas bag and analyzed by gas chromatography (GC, HP 4890D), which was equipped with TCD detectors using argon as the carrier gas. The liquid product was analyzed by 1 H NMR (Bruker Avance III 400 HD spectrometer) in deuteroxide.

Calculations of Faradaic efficiencies of gaseous and liquid products.

Liquid products:

After electrolysis, a certain amount of internal standard solution was added to the electrolyte as the internal standard. Because the concentration of internal standard was known, the moles of liquid products can be calculated from integral areas and calibration curves. To accurately integrate the products in NMR analysis, two standard peaks located in different regions were used in NMR analysis. The sodium 2, 2-dimethyl-2-silapentane-5-sulfonate (DSS) was the reference for n-propanol, ethanol and acetic acid. And the phenol was the reference for formate. 400 μL catholyte after the reaction was mixed with 100 uL 6 mM DSS solution, 100 μL 200 mM phenol and 200 μL D_2O, and then analyzed by 1H NMR (Bruker Avance III 400 HD spectrometer).

The Faradaic efficiency of liquid product is[S4][S5]:

$$FE = \frac{moles\ of\ products}{Q/nF} \times 100\ \%$$

(Q: Charge; F: Faradaic constant; n: transfer electron number)

Gaseous products:

From the GC peak areas and calibration curves for the TCD detector, we can obtain the V % of gaseous products. Since the flow rate of the CO_2 was constant, the moles of gaseous products can be calculated. The Faradaic efficiency of gaseous product is:

$$FE = \frac{moles\ of\ products}{Q/nF} \times 100\ \%$$

(Q: Charge; F: Faradaic constant; n: transfer electron number)

Computational Details:

All the density functional theory (DFT) calculations were carried out using the Vienna *ab initio* Simulation Package (VASP ver. 5.4.4).[6-8] The interactions between electronics and ions was described using the projector augmented wave (PAW) method.[9] The plane wave cutoff was set to 400 eV. The exchange-correlation potential was calculated by the generalized gradient approximation (GGA) with the Perdew-Burke-Ernzerhof (PBE) functional.[10] The Brillouin zone sampling was performed on meshes with a k-point spacing of 0.06 $Å^{-1}$ for Cu/NC and 0.03 $Å^{-1}$ for Cu(111) surface.[11] The energy criterion is set to 10^{-5} eV in iterative solution of the Kohn-Sham equation. The geometry optimization convergences tolerance was set to 0.05 eV $Å^{-1}$. The graphene calculation model was made of an 8*6 lattice supercell. Some of C atoms were replaced to N to investigate the role of N doping atoms in NC and Cu/NC. The Cu cluster in Cu-CN was considered as 4 layers of Cu with the surface of (111).[12] The slab model with 4*4 lattice supercell was built as the calculation model of Cu(111) surface. The Cu atoms in the bottom two layers were fixed, and the top two layers were fully relaxed. The energy of H^+ was considered as half of the energy of a hydrogen molecule to correspond to the equilibrium equation of 1/2 H_2 <==>H^+ at 0 V vs. RHE. The geometrical configurations were illustrated with VESTA software.[13]

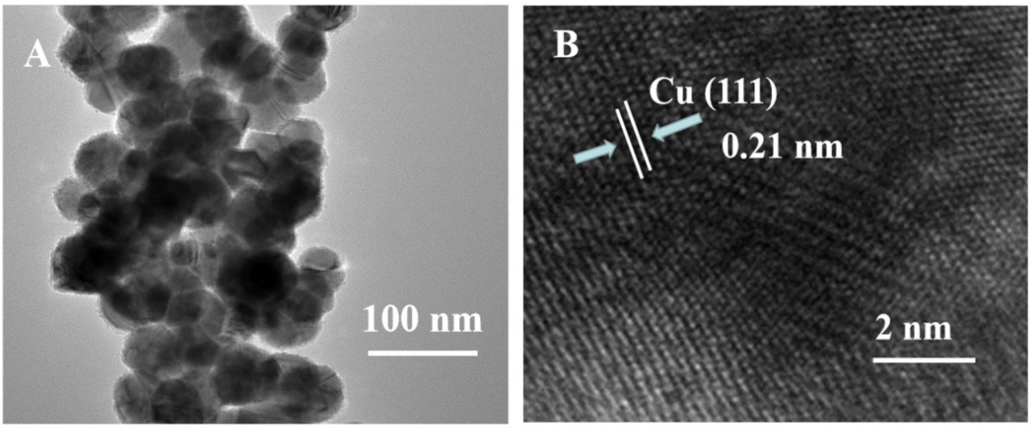

Figure S1. TEM (A) and HR-TEM (B) images of Cu-np.

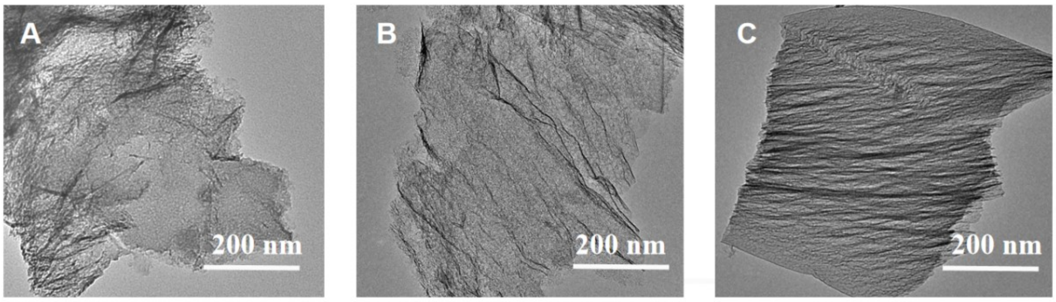

Figure S2. TEM images of NC(1:2) (A), NC(1:4) (B) and NC(1:8) (C).

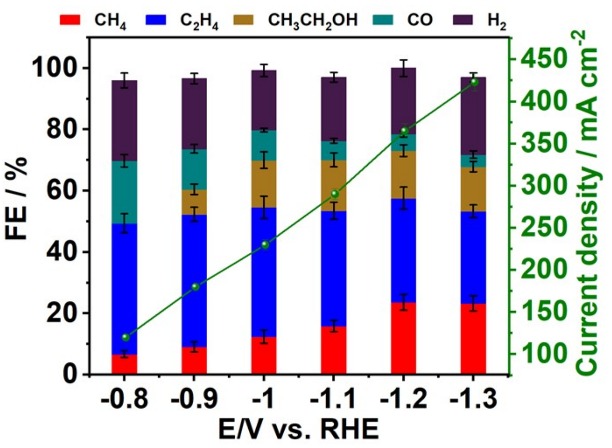

Figure S3. The faradaic efficiency and current density of Cu-np at various applied potentials in CO_2RR.

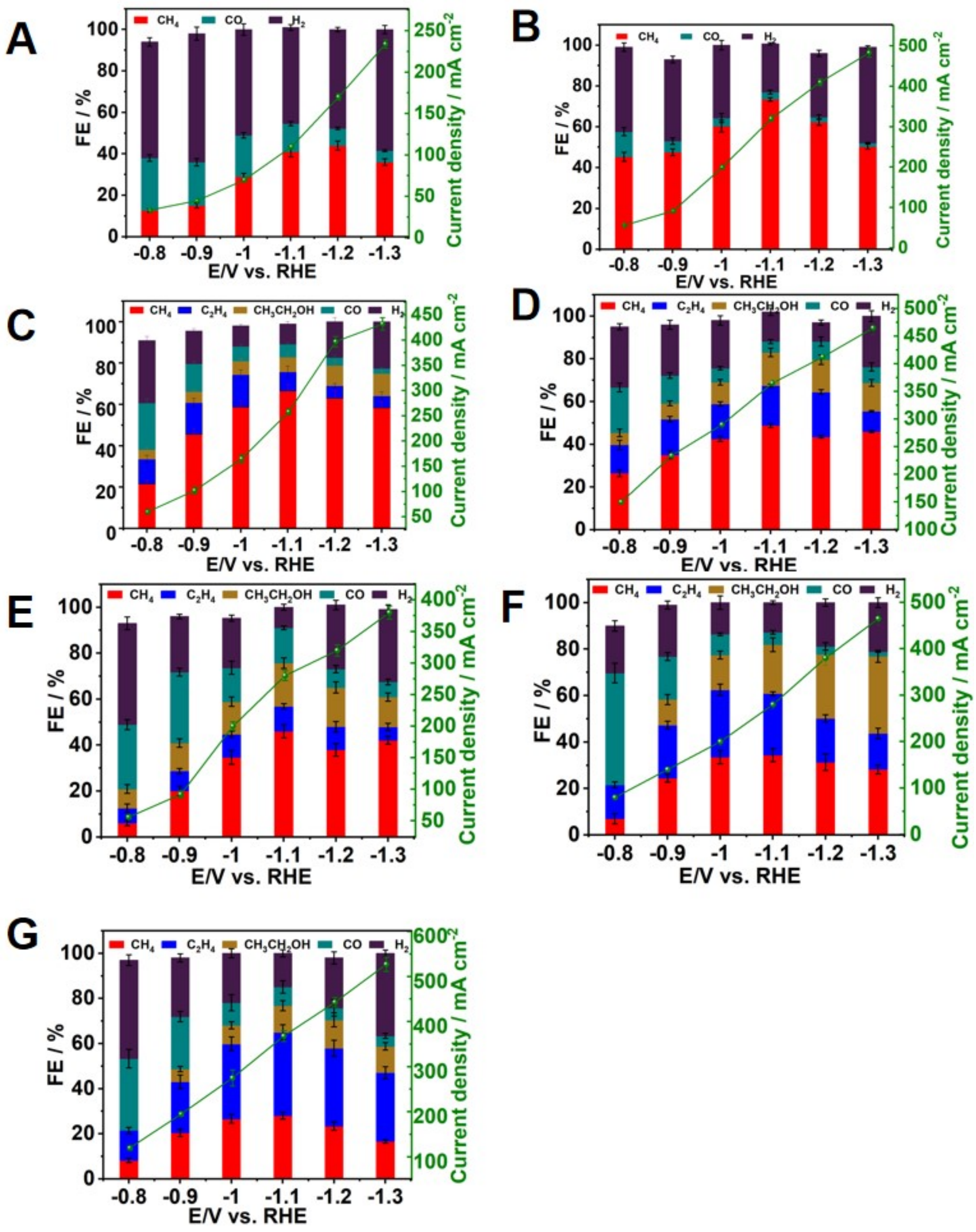

Figure S4. The faradaic efficiency and current density of Cu-np/NC(1:4) with different content of Cu-np at various applied potentials in CO_2RR. (A) Cu-np/NC (1:4, 99.5 wt%); (B) Cu-np/NC (1:4, 99 wt%); (C) Cu-np/NC (1:4, 98.5 wt%); (D) Cu-np/NC (1:4, 97 wt%); (E) Cu-np/NC (1:4, 95 wt%); (F) Cu-np/NC (1:4, 80 wt%); (G) Cu-np/NC (1:4, 70 wt%).

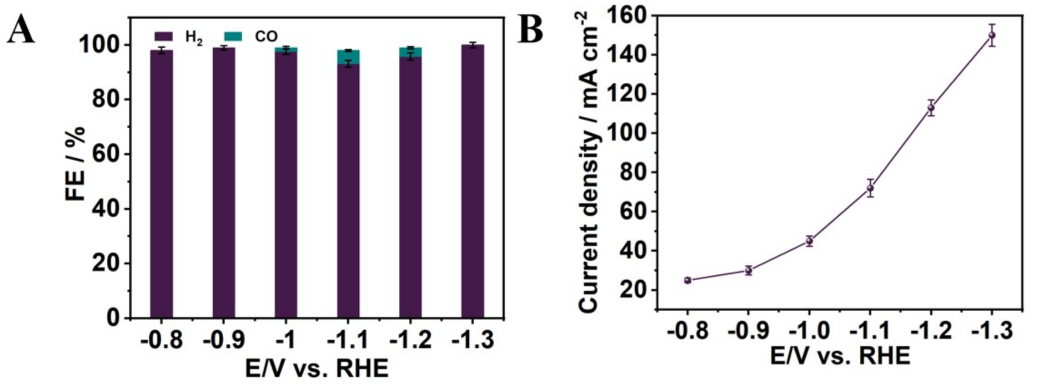

Figure S5. (A) The faradaic efficiency over NC(1:4) at various applied potentials in CO_2RR. (B) The current density over NC(1;4) at various applied potentials in CO_2RR.

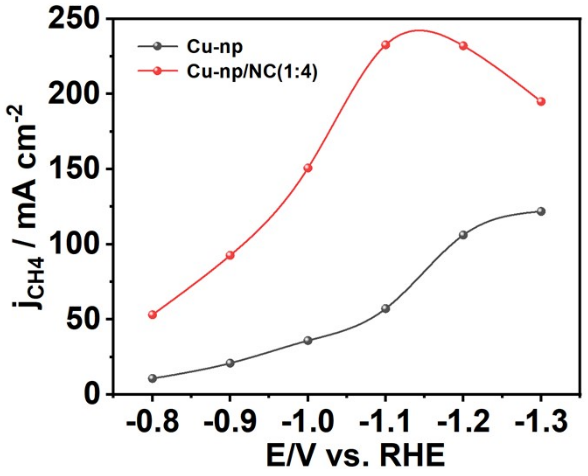

Figure S6. The partial current density of CH_4 over different catalysts.

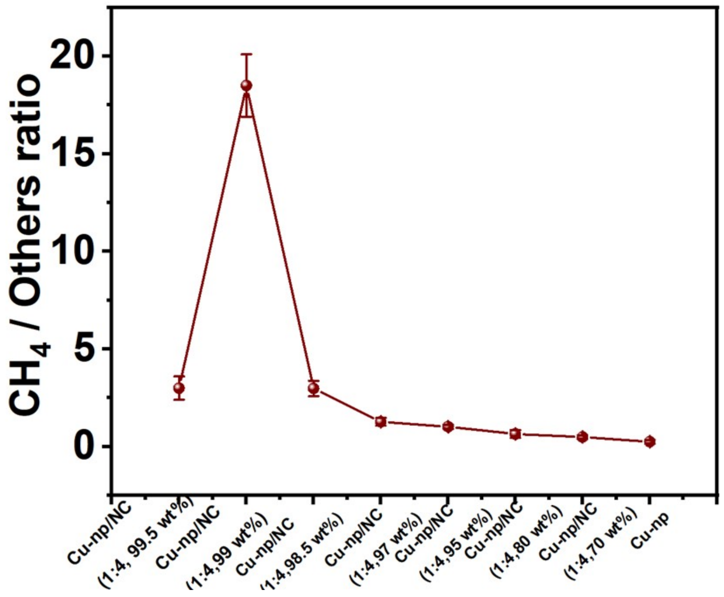

Figure S7. The CH_4-to-others ratio for Cu-np and Cu-np/NC(1:4).

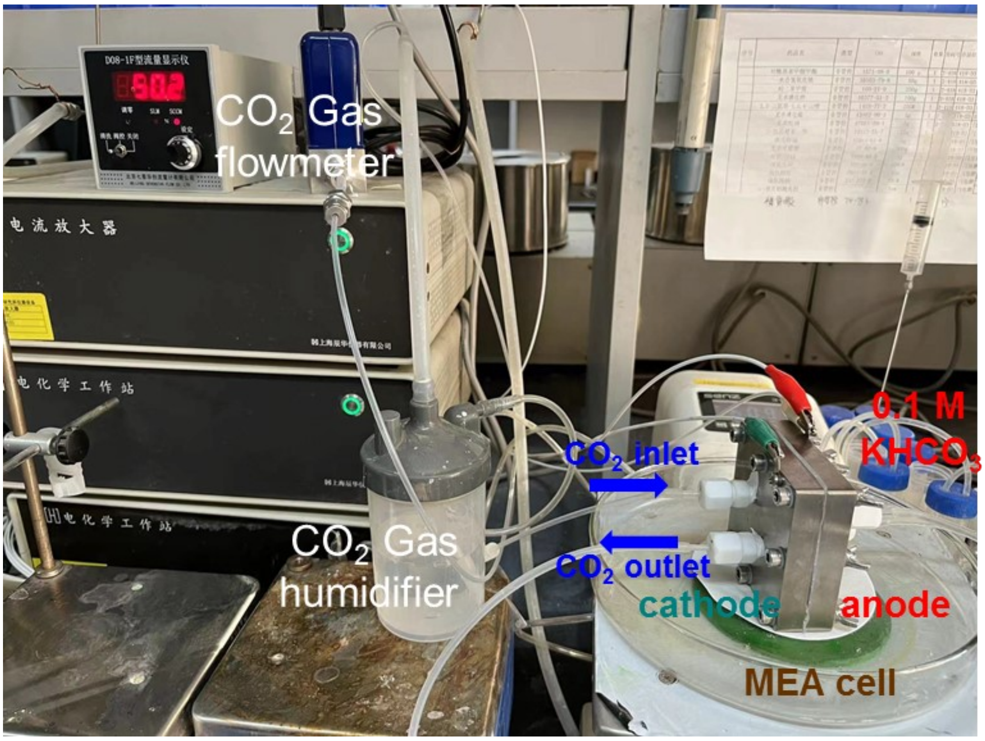

Figure S8. The Membrane electrode assembly-based reactors for CO_2RR.

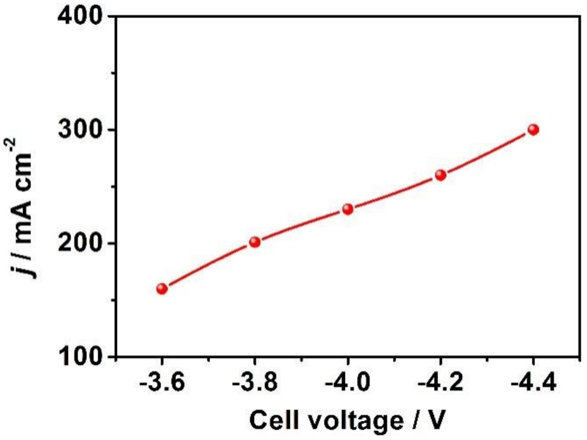

Figure S9. The current density over Cu-np/NC(1:4) for CO_2RR in MEA.

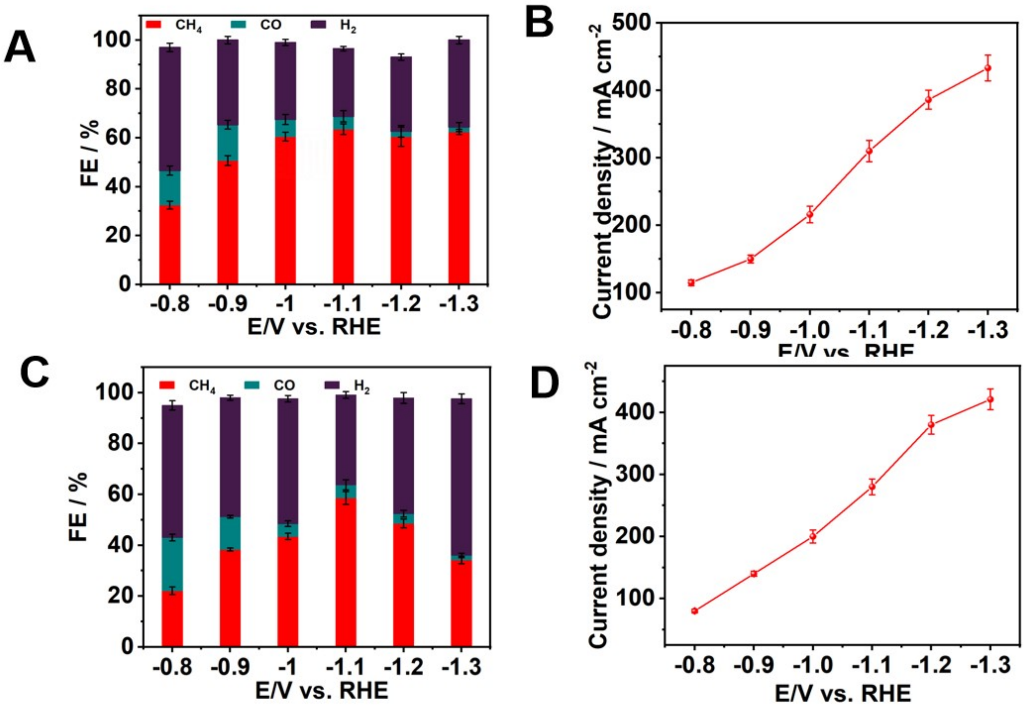

Figure S10. The faradaic efficiency and current density over Cu-np/NC(x:y) with different x:y values at various applied potentials in CO_2RR. (A, B) Cu-np/NC(1:2); (C, D) Cu-np/NC (1:8).

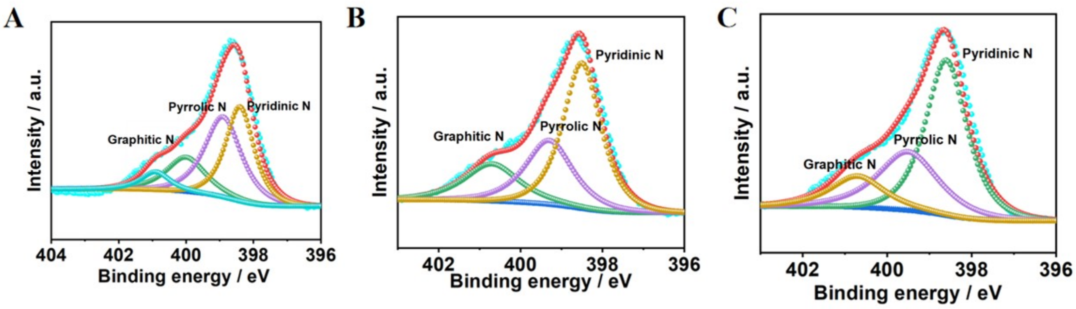

Figure S11. The N 1s XPS spectra of NC (1:2) (A), NC (1:4) (B) and NC (1:8) (C).

.

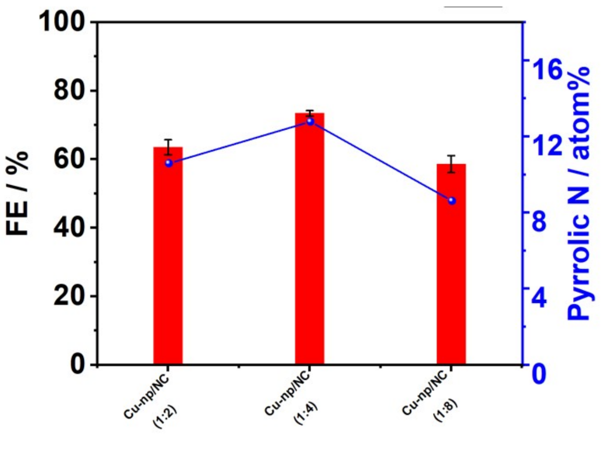

Figure S12. The effect of pyrrolic N content of Cu-np/NC(x:y) on the FE of CH_4.

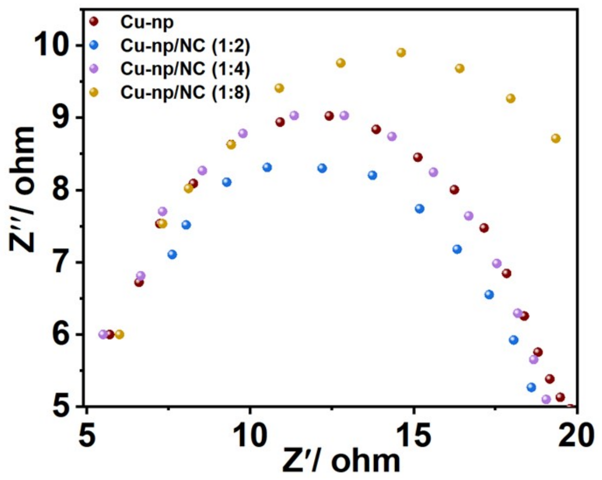

Figure S13. Nyquist plots for different electrodes.

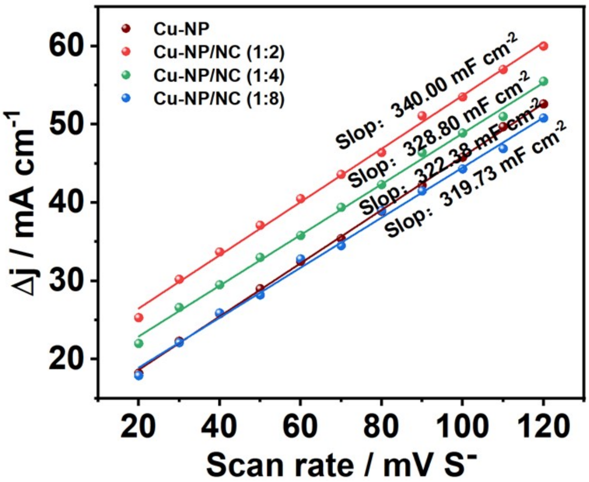

Figure S14. The charging current density differences plotted against the scan rates for different catalysts.

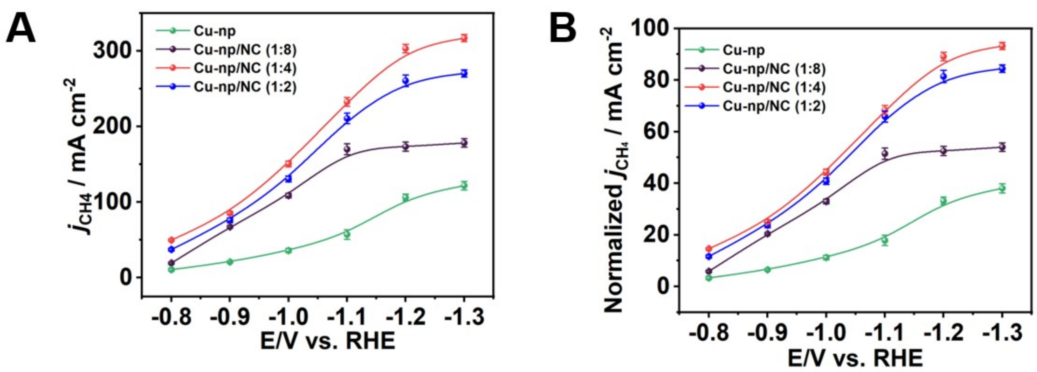

Figure S15. (A) The partial current density of CH_4 for Cu-np/NC(x:y); (B) The normalized partial current density for CH_4 by ECSAs for Cu-np/NC(x:y).

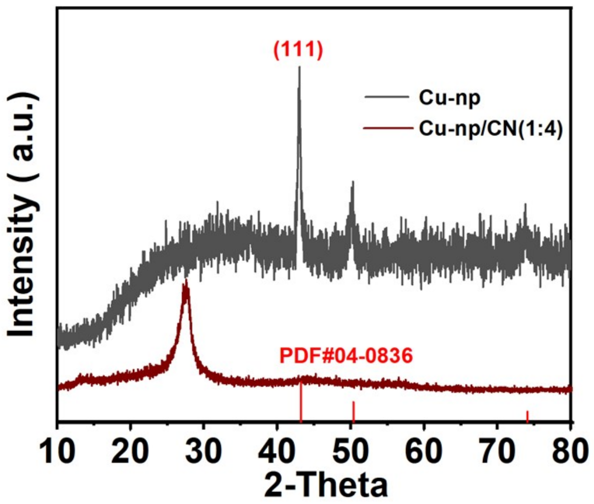

Figure S16.XRD patterns of Cu-np and Cu-np/CN(1:4) samples.

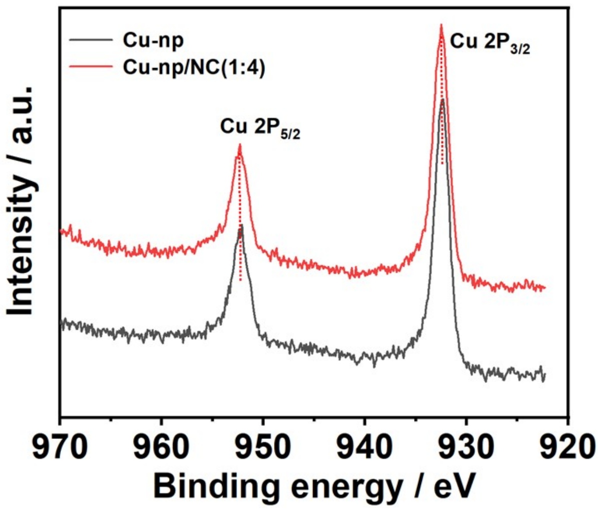

Figure S17.The Cu 2p XPS spectra of Cu-np and Cu-np/NC(1:4).

.

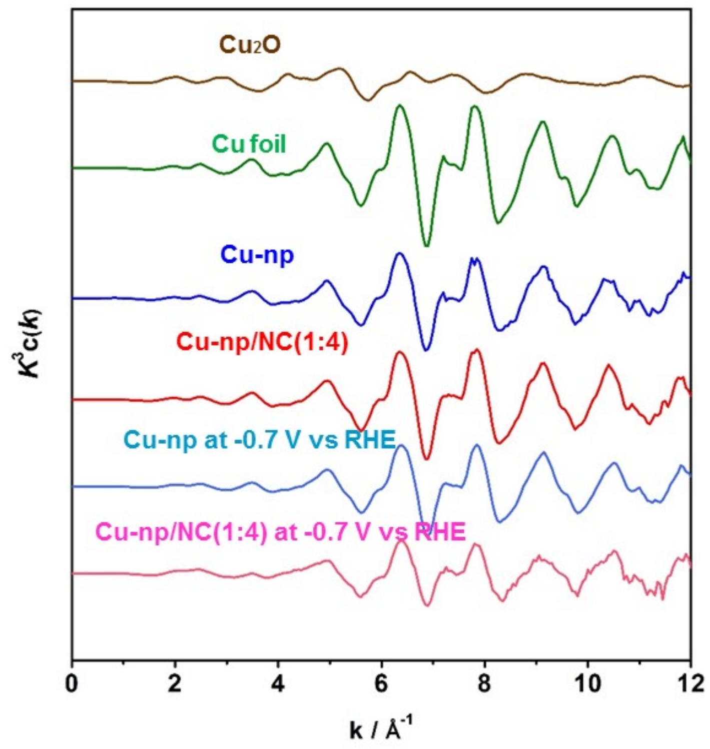

Figure S18. Operando Cu K-edge extended XAFS oscillation function $k^3w(k)$ over different catalysts.

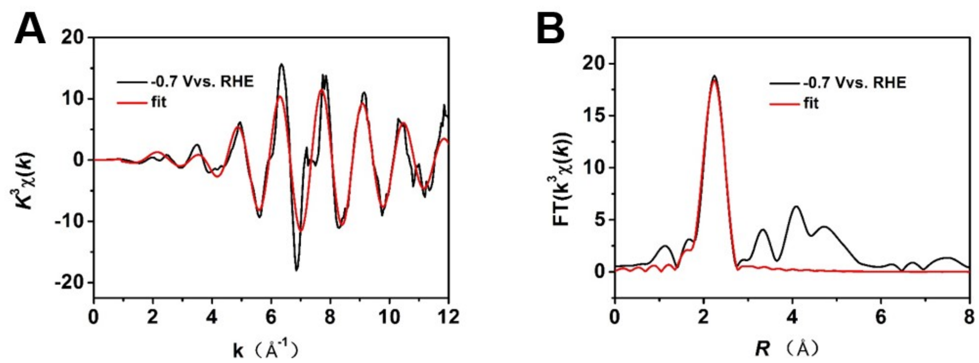

Figure S19. (**A**) Cu K-edge EXAFS fitting analysis of Cu-np in *k* space. (**B**) Cu K-edge EXAFS fitting analysis of Cu-np in *R* space.

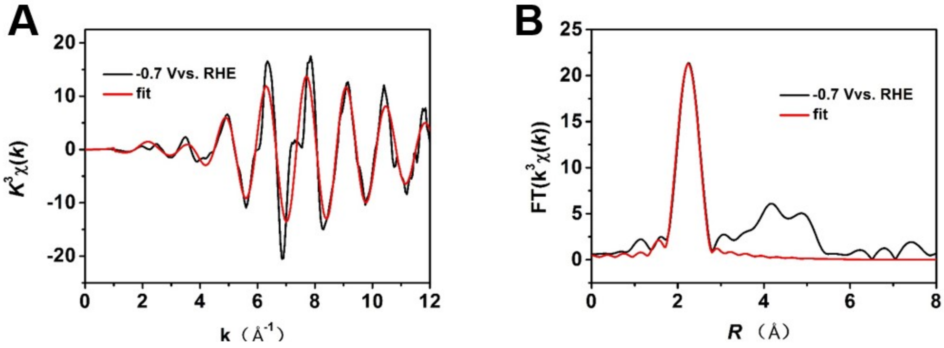

Figure S20. (**A**) Cu K-edge EXAFS fitting analysis of Cu-np/NC(1:4) in *k* space. (**B**) Cu K-edge EXAFS fitting analysis of Cu-np/NC(1:4) in *R* space.

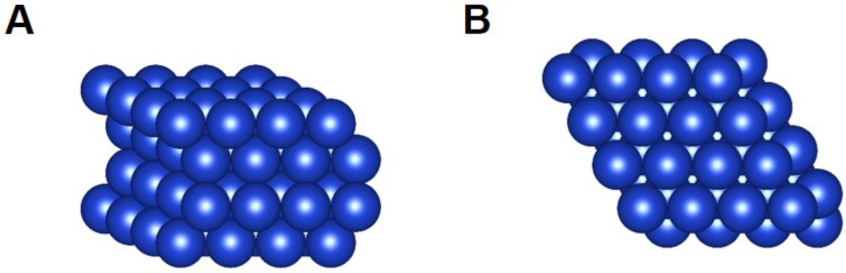

Figure S21. (A) Side view and (B) top view of the Cu (111).

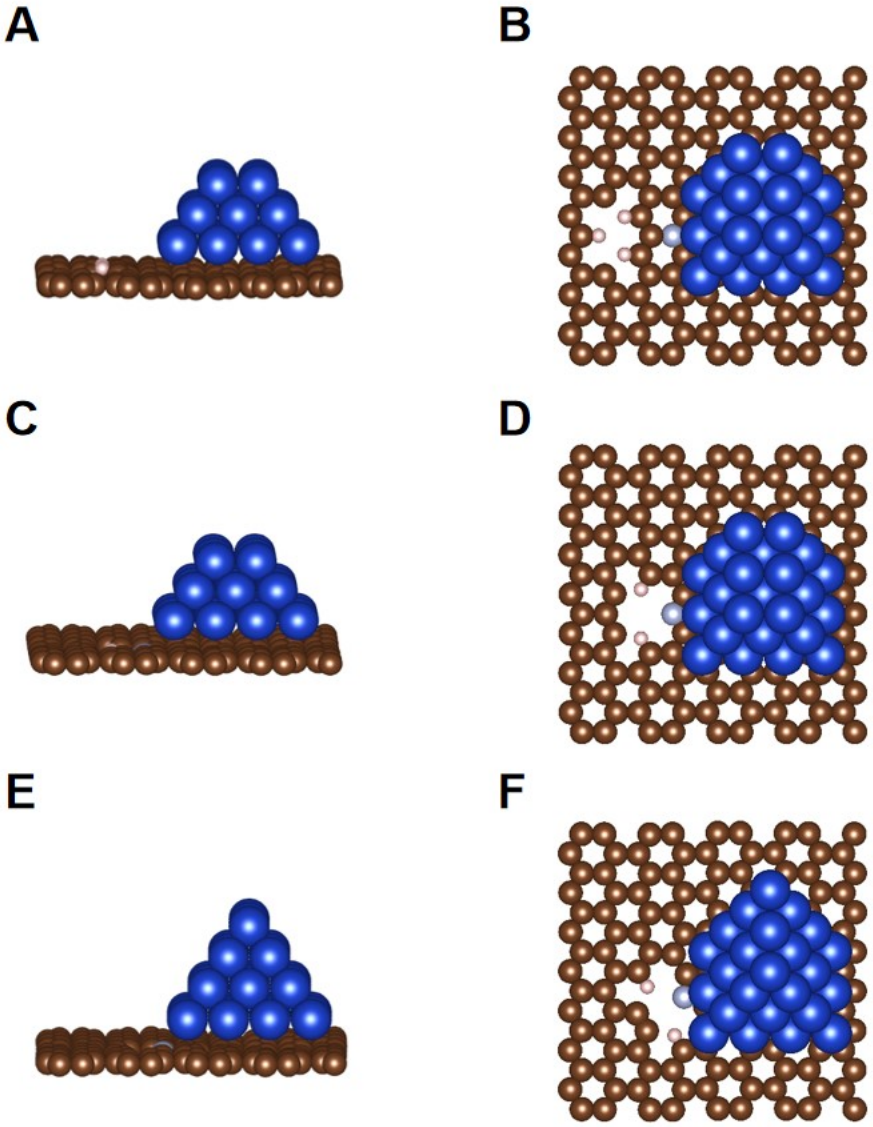

Figure S22. (A) Side view and (B) top view of the Cu(111)/ Graphitic N. (C) Side view and (D) top view of the Cu (111)/Pyridinic N. (E) Side view and (F) top view of the Cu(111) Pyrrolic N.

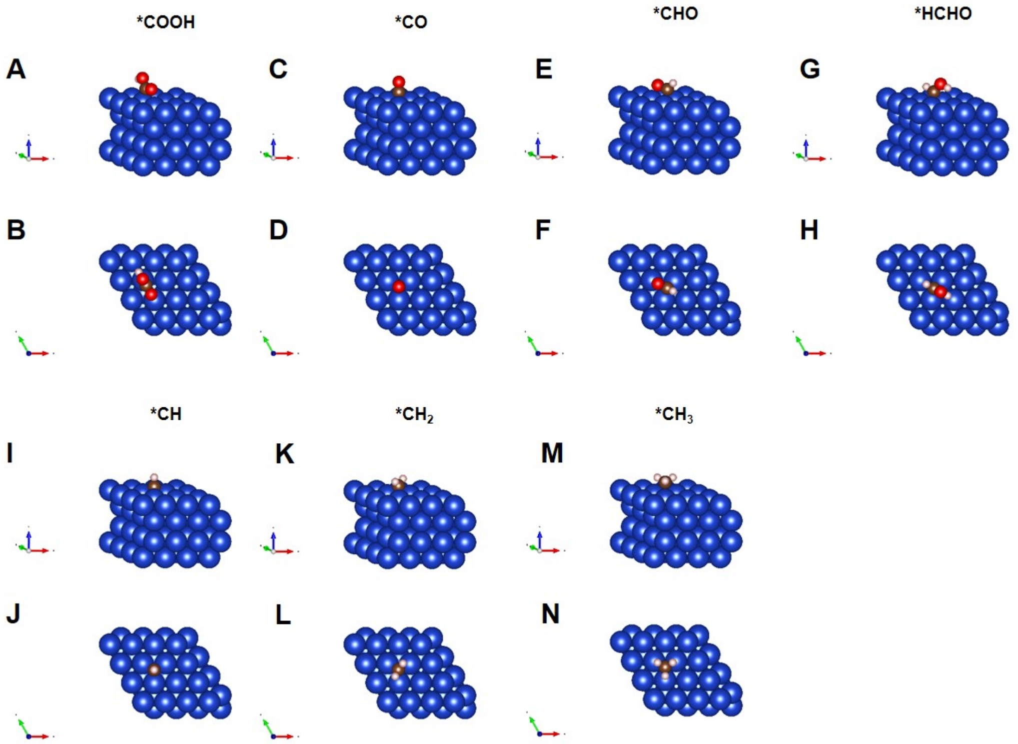

Figure S23. (A) Side view and (B) top view of *COOH on the Cu (111). (C) Side view and (D) top view of *CO on the Cu (111). (E) Side view and (F) top view of *CHO on the Cu (111). (G) Side view and (H) top view of *HCHO on the Cu (111). (I) Side view and (J) top view of *CH on the Cu (111). (K) Side view and (L) top view of $*CH_2$ on the Cu (111). (M) Side view and (N) top view of $*CH_3$ on the Cu (111). The atoms in blue, brown, red, and pink represent Cu, C, O, and H, respectively.

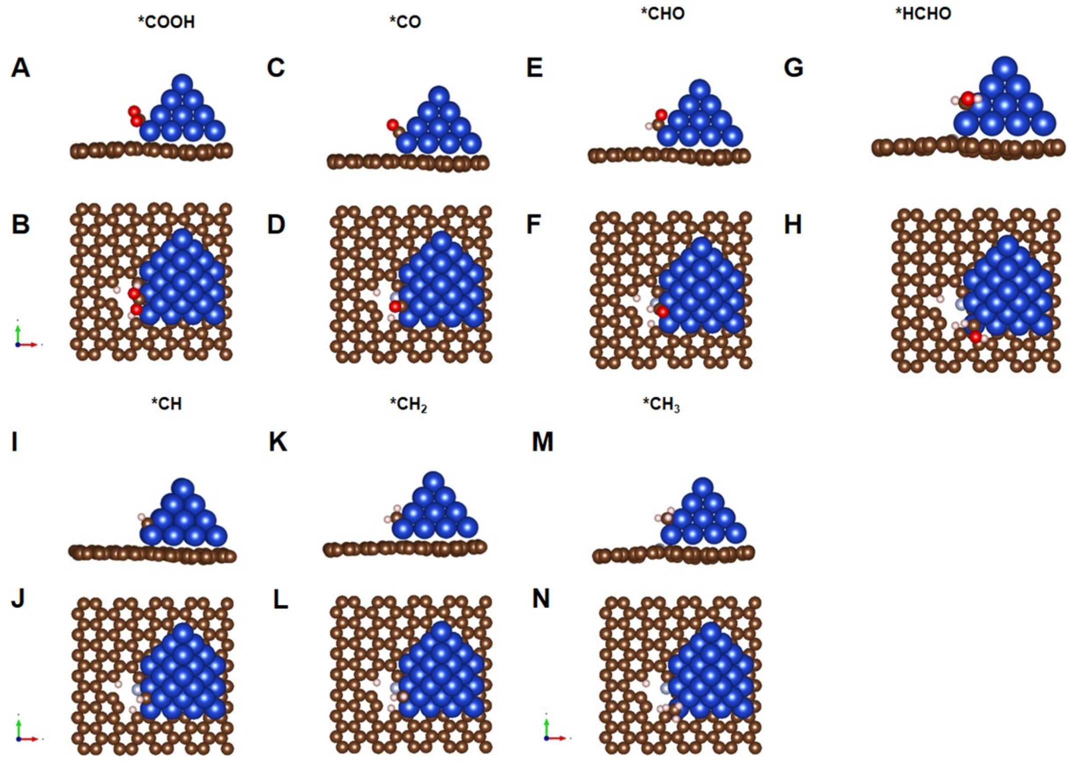

Figure S24. (A) Side view and (B) top view of *COOH on the Cu (111)/Pyrrolic N. (C) Side view and (D) top view of *CO on the Cu (111) /Pyrrolic N. (E) Side view and (F) top view of *CHO on the Cu (111) /Pyrrolic N. (G) Side view and (H) top view of *HCHO on the Cu (111) /Pyrrolic N. (I) Side view and (J) top view of *CH on the Cu (111) /Pyrrolic N. (K) Side view and (L) top view of *CH_2 on the Cu (111) /Pyrrolic N. (M) Side view and (N) top view of *CH_3 on the Cu (111) /Pyrrolic N. The atoms in blue, brown, red, and pink represent Cu, C, O, and H, respectively.

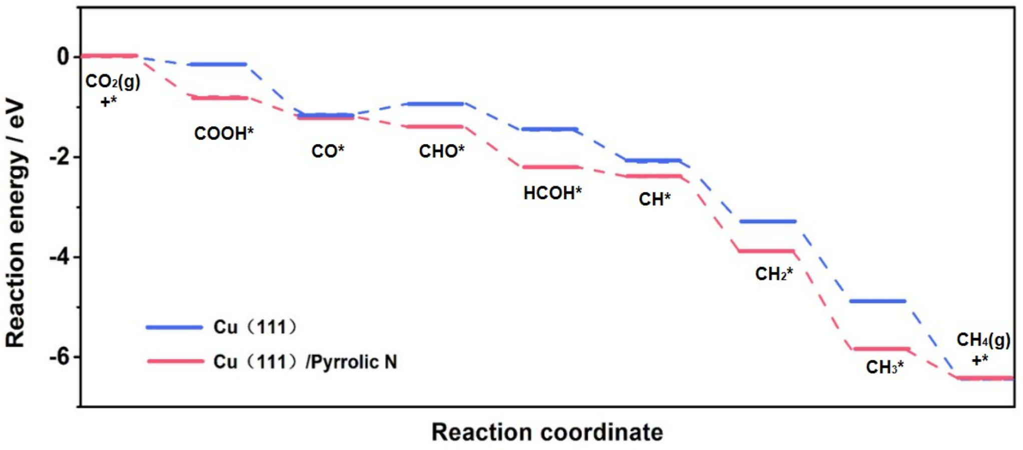

Figure S25. A reaction energy diagram for the CO_2RR to CH_4 over Cu(111) and Cu(111)/Pyrrolic N at -0.5 V.

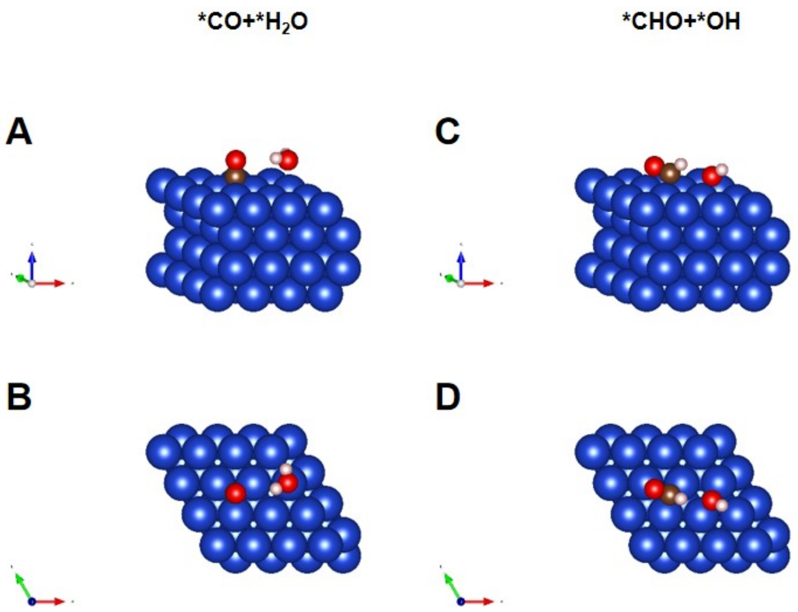

Figure S26. (A) Side view and (B) top view of *CO and *H_2O on the Cu (111). (C) Side view and (D) top view of *CHO and *OH on the Cu (111).

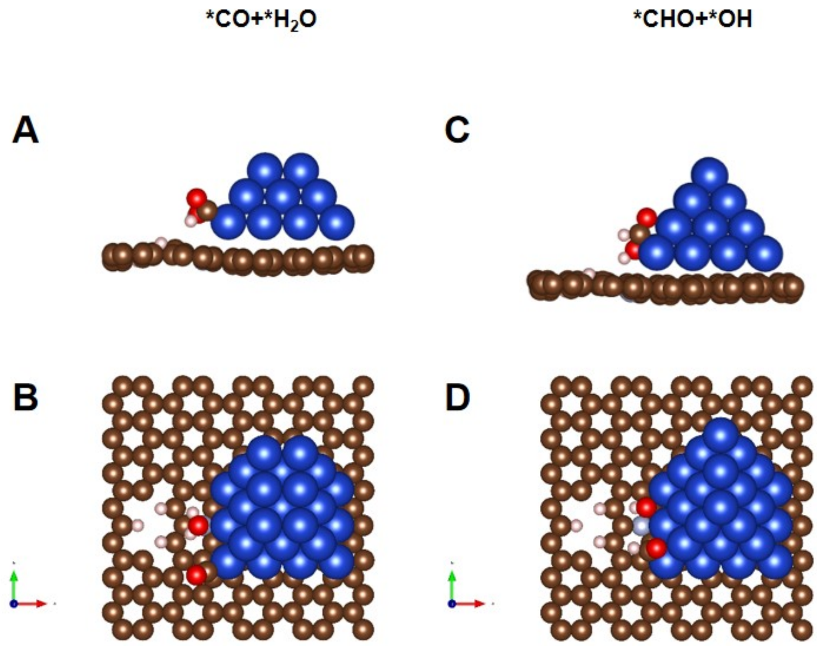

Figure S27. (A) Side view and (B) top view of *CO and *H_2O on the Cu(111)/ Graphitic N. (C) Side view and (D) top view of *CHO and *OH on the Cu(111)/ Graphitic N.

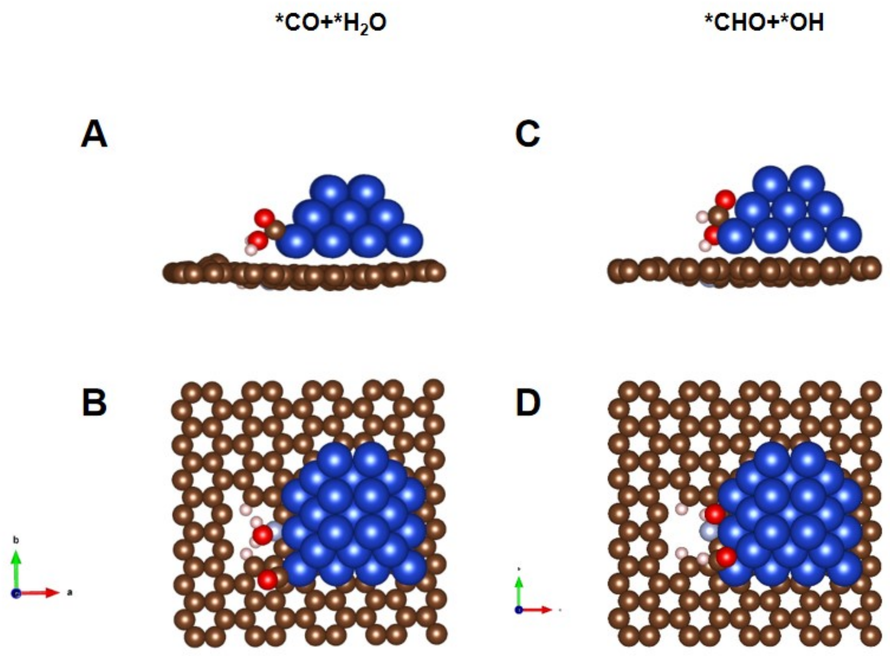

Figure S28. (A) Side view and (B) top view of *CO and *H_2O on the Cu (111)/Pyridinic N. (C) Side view and (D) top view of *CHO and *OH on the Cu (111)/Pyridinic N.

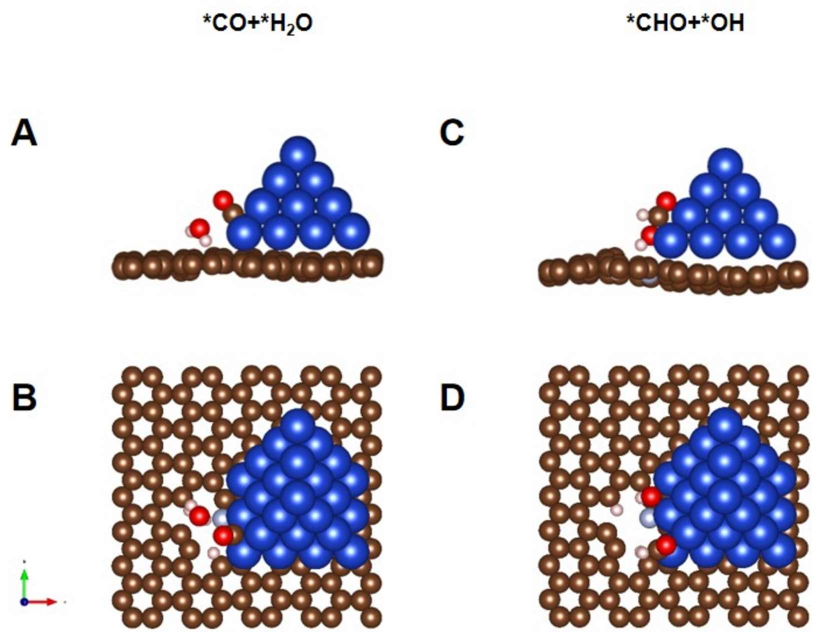

Figure S29. (A) Side view and (B) top view of *CO and *H_2O on the Cu (111)/Pyrrolic N. (C) Side view and (D) top view of *CHO and *OH on the Cu (111)/Pyrrolic N.

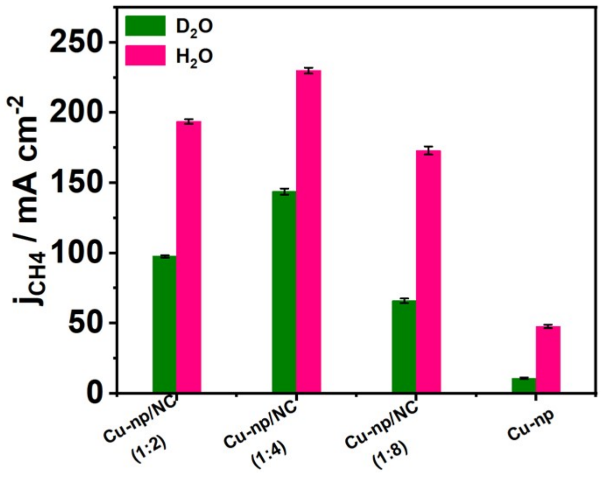

Figure S30. Partial current density of CH_4 in H_2O and D_2O over Cu-np/NC(x:y) and Cu-np catalyst. The reactions were conducted at -1.1 V versus RHE in 1.0 M KOH.

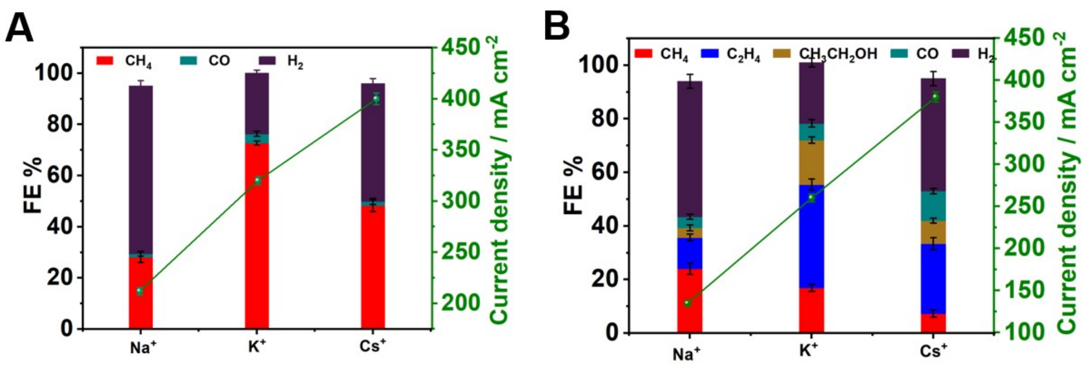

Figure S31. Effect of alkali metal cation in MOH on HER and CO_2RR. MOH (M = Na^+, K^+ and Cs^+) was used as electrolyte. (A) FEs of all products and current density for CO_2RR over Cu-np/NC(1:4) catalyst. (B) FEs of all products and current density or CO_2RR over Cu-np catalyst. The reactions were conducted at −1.1 V versus RHE in 1.0 M MOH (M =Na^+, K^+ and Cs^+) electrolytes.

Table S1. FE and current density comparison of various Cu base elctrocatalysts for CO_2 electroreduction to CH_4.

Catalysts	Electrolyte	Potential / V	FE / %	Current density / mA cm^{-2}	Refs.
Cu-np/NC (1:4)	1 M KOH	-1.1 V vs. RHE	73.4±5	320±10	This work
Ag@Cu_2O - 6.4 NCs	1 M KOH	-1.2 V vs. RHE	74±2	178±5	S14
Cu_2O@ CuHHTP	0.1 M KCl/0.1 M $KHCO_3$	-1.4 V vs. RHE	73	10.8	S15
Cu_2O@ HKUST-1	0.1 M $KHCO_3$	-1.71 V vs. RHE	63.2	8.2	S16
Cu-N-C	0.1 M $KHCO_3$	-1.6 V vs. RHE	38.6	14.8	S17
La_2CuO_4	1 M KOH	-1.4 V vs. RHE	56.3	117	S18
Cu_{oh}	1 M KOH	-0.91 V vs. RHE	53	100	S19
Cu-MOF-74	0.1 M KCO_3	-1.3 V vs. RHE	50	3..8	S20
Pluse electrodeposit ed Cu	0.5 M $NaHCO_3$	-2.8 V vs. SCE	85	38	S21
Cu clusters/ DRC	0.1 M $KHCO_3$	-1 V vs. RHE	81.7	18	S22
Cu foil	0.5 M $NaHCO_3$	-1.98 V vs. RHE	81.6	31	S23
n-Cu/C	0.1 M $NaHCO_3$	-1.35 V vs. RHE	76	7	S24
CuS	0.1 M $KHCO_3$	-1.1 V vs. RHE	73	6	S25
Cu-TDPP-NS	0.5 M PBS	-1.6 V vs. RHE	70	183	S26
CoO/Cu/PTFE	1 M KOH	-1.1 V vs. RHE	60	225	S27
Cu-DBC	1 M KOH	-0.9 V vs. RHE	80	203	S28

Cu-3TPyP	1 M KOH	-1.6 V vs. RHE	62.4	229	S29
CoO/Cu/PTFE	1.0 M $KHCO_3$	-1.1 V vs. RHE	60	135	S30
Cu-CeO_2	0.1 M KHCO3	-1.8 V vs. RHE	58	33.6	S31
CuNWs@PDA	0.5 M KHCO3	-0.93 V vs. RHE	30	2.1	S32
Cu NPs	0.1 M KHCO3	-1.1 V vs. RHE	20	0.5	S33
Cu/p-Al2O3 SAC	1.0 M KOH	-1.2 V vs. RHE	62	94.8	S34
NNU-33(H)	1.0 M KOH	-0.9 V vs. RHE	82	390	S35
Cu	0.5 M NaHCO3	-2.2 V vs. RHE	85	38	S36

Table S2. The content of N in different catalysts.

Catalysts	The content N (atom %)	The pyrrolic N (atom %)	The pyridinic N (atom %)	The graphitic N (atom %)
NC (1:2)	33.4	10.6	15.9	6.9
NC (1:4)	45.8	12.8	25.6	7.4
NC (1:8)	42.9	8.6	26.0	8.3

Table S3 Structural parameters of Cu-np and Cu-np/NC(1:4) at -0.7 V vs RHE during CO_2RR extracted from the EXAFS fitting. (S_0^2=0.80)

Sample	Scattering pair	CN	R(Å)	$\sigma^2(10^{-3}Å^2)$	ΔE_0(eV)
Cu-np	Cu-Cu	9.3±0.6	2.55±0.02	5.8±0.6	3.3±0.3
Cu-np/NC(1:4)	Cu-Cu	9.8±0.7	2.55±0.02	5.8±0.8	3.3±0.4

S_0^2 is the amplitude reduction factor S_0^2=0.8; CN is the coordination number; R is interatomic distance (the bond length between central atoms and surrounding coordination atoms); σ^2 is Debye-Waller factor (a measure of thermal and static disorder in absorber-scatterer distances); ΔE_0 is edge-energy shift (the difference between the zero kinetic energy value of the sample and that of the theoretical model). R factor is used to value the goodness of the fitting.

References

S1 C. Choi, T. Cheng, M. Flores Espinosa, H. Fei, X. Duan, W. A. Goddard III, Y. Huang, *Adv. Mater.* **2019**, *31*, e1805405.

S2 L. Li, C. Tang, Y. Zheng, B. Xia, X. Zhou, H. Xu, S. Qiao, *Adv. Energy. Mater.* **2020**, *10*.2000789.

S3 F. Li, A. Thevenon, A. Rosas-Hernandez, Z. Wang, Y. Li, C. M. Gabardo, A. Ozden, C. T. Dinh, J. Li, Y. Wang, J.P. Edwards, Y. Xu, C. McCallum, L. Tao, Z.-Q Liang, M. Luo, X. Wang, H. Li, C. P. O'Brien, C.-S. Tan, D.-H.Nam, R. Quintero-Bermudez, T.-T. Zhuang, Y.C. Li, Z. Han, R. D. Britt, D. Sinton, T. Agapie, J.C. Peters, E. H.Sargent, *Nature.* **2020**, 577, 509-513.

S4 D. Yang, Q. Zhu, X. Sun, C. Chen, W. Guo, G. Yang, B. Han, *Angew. Chem. Int. Ed.* **2019**,*132*,2374-2379.

S5 Y. Wu, C. Chen, X. Yan, S. Liu, M. Chu, H. Wu, J. Ma and B. Han, *Green. Chem.***2020**，*22*， 6340-6344.

S6 G. Kresse, D. Joubert, *Phys. Rev. B.* **1999**, *59*, 1758.

S7 G. Kresse, J. Furthmüller, *Phys. Rev. B.* **1996**, *54*, 11169.

S8 G. Kresse, J. Furthmüller, *Comput. Mater. Sci*. **1996**, *6*, 15.

S9 P. E. Blöchl, *Phys. Rev. B* .**1994**, *50*, 17953.

S10 J. P. Perdew, K. Burke, M. Ernzerhof, *Phys. Rev. Lett*. **1996**, *77*, 3865.

S11 H. J. Monkhorst, J. D. Pack, *Phys. Rev. B.* **1976**, *13*, 5188.

S12 J. Wu, M. Liu, P. P. Sharma, R. M. Yadav, L. Ma, Y. Yang, X. Zou, X. D. Zhou, R. Vajtai, B. I. Yakobson, J. Lou, P. M. Ajayan, *Nano. Lett.* **2016**, *16*, 466-470.

S13 K. Momma, F. J. Izumi. Appl. Crystallogr. **2011**, *44*, 1272.

S14 L. Xiong, X. Zhang, L. Chen, Z. Deng, S. Han, Y. Chen, J. Zhong, H. Sun, Y. Lian, B. Yang, X. Yuan, H. Yu, Y. Liu, X. Yang, J. Guo, M. H. Rummeli, Y. Jiao, Y. Peng, *Adv. Mater.* **2021**, *33*, e2101741.

S15 J. D. Yi, R. Xie, Z. L. Xie, G. L. Chai, T. F. Liu, R. P. Chen, Y. B. Huang, R. Cao, *Angew. Chem. Int. Ed.* **2020**, *59*, 23641-23648.

S16 X. Tan, C. Yu, C. Zhao, H. Huang, X. Yao, X. Han, W. Guo, S. Cui, H. Huang, J. Qiu, *ACS. Appl. Mater. Inter.* **2019**, *11*, 9904-9910.

S17 A. Guan, Z. Chen, Y. Quan, C. Peng, Z. Wang, T.-K. Sham, C. Yang, Y. Ji, L. Qian, X. Xu, G. Zheng, *ACS. Energy. Lett.* **2020**, *5*, 1044-1053.

S18 S. Chen, Y. Su, P. Deng, R. Qi, J. Zhu, J. Chen, Z. Wang, L. Zhou, X. Guo, B. Y. Xia, *ACS. Catal.* **2020**, *10*, 4640-4646.

S19 G. L. De Gregorio, T. Burdyny, A. Loiudice, P. Iyengar, W. A. Smith, R. Buonsanti, *ACS. Catal.* **2020**, *10*, 4854-4862.

S20 M. K. Kim, H. J. Kim, H. Lim, Y. Kwon, H. M. Jeong, *Electrochim. Acta.* **2019**, 306,28-34.

S21 Y.-L. Qiu, H.-X. Zhong, T.-T. Zhang, W.-B. Xu, X.-F. Li, H.-M. Zhang, *ACS. Catal.* **2017**,*7*, 6302-6310.

S22 Q. Hu, Z. Han, X. Wang, G. Li, Z. Wang, X. Huang, H. Yang, X. Ren, Q. Zhang, J. Liu, C. He, *Angew. Chem. Int. Ed.* **2020**, *59*, 19054-19059.

S23 Y. Qiu, H. Zhong, W. Xu, T. Zhang, X. Li, H. Zhang, *J. Mater. Chem. A.* **2019**, *7*, 5453-

5462.

S24 K. Manthiram, B.J. Beberwyck, A. P. Alivisatos, *J. Am. Chem. Soc*. **2014**, *136*, 13319-13325.

S25 Z. Zhao, X. Peng, X. Liu, X. Sun, J. Shi, L. Han, G. Li, J. Luo, *J. Mater. Chem. A*. **2017**, *5*, 20239-20243.

S26 Y.-R. Wang, M. Liu, G.-K. Gao, Y.-L. Yang, R.-X. Yang, H.-M. Ding, Y. Chen, S.-L. Li, Y.-Q. Lan, *Angew. Chem. Int. Ed.* **2021**.*27*,22123-22129.

S27 Y. Li, A. Xu, Y. Lum, X. Wang, S.-F. Hung, B. Chen, Z. Wang, Y. Xu, F. Li, J. Abed, J. E. Huang, A. S. Rasouli, J. Wicks, L. K. Sagar, T. Peng, A. H. Ip, D. Sinton, H. Jiang, C. Li, E. H. Sargent, *Nat. Commun.* **2020**, *11*, 6190.

S28 Y. Zhang, L.-Z. Dong, S.-Li, X. Huang, J.-N. Chang, J.-H. Wang, J. Zhou, S.-L. Li, Y.-Q. Lan, *Nat. Commun.* **2021**, *12*, 6390.

S29 S.-N. Sun, J.-N. Lu, Q. Li, L.-Z. Dong, Q. Huang, J. Liu, Y.-Q. Lan, *Chem. Catal.* **2021**, *1*, 1133-1144.

S30 Y. Li, A. Xu, Y. Lum, X. Wang, S.-F. Hung, B. Chen, Z. Wang, Y. Xu, F. Li, J. Abed, J. E. Huang, A. S. Rasouli, J. Wicks, L. K. Sagar, T. Peng, A. H. Ip, D. Sinton, H. Jiang, C. Li and E. H. Sargent, *Nat. Commun*, **2020**, *11*, 6190.

S31 B. Zhou, H. Sato, L. Ilies and E. Nakamura, *ACS. Catal*, **2018**, *8*, 8-11.

S32 H. Liu, K. Xiang, Y. Liu, F. Zhu, M. Zou, X. Yan and L. Chai, *ChemElectroChem*, **2018**, *5*, 3991-3999.

S33 J. Resasco, L. D. Chen, E. Clark, C. Tsai, C. Hahn, T. F. Jaramillo, K. Chan and A. T. Bell, *J. Am. Chem. Soc*, **2017**, *139*, 11277-11287.

S34 S. Chen, B. Wang, J. Zhu, L. Wang, H. Ou, Z. Zhang, X. Liang, L. Zheng, L. Zhou, Y.-Q. Su, D. Wang and Y. Li, *Nano Lett*, **2021**, *21*, 7325-7331.

S35 L. Zhang, X.-X. Li, Z.-L. Lang, Y. Liu, J. Liu, L. Yuan, W.-Y. Lu, Y.-S. Xia, L.-Z. Dong, D.-Q. Yuan and Y.-Q. Lan, *J. Am. Chem. Soc*, **2021**, *143*, 3808-3816.

S36 Y.-L. Qiu, H.-X. Zhong, T.-T. Zhang, W.-B. Xu, X.-F. Li and H.-M. Zhang, *ACS Catal*, **2017**, *9*, 6302–6310.
